# Effects of cryptotanshinone in treatment of polycystic ovary syndrome in rats: a systematic review and meta-analysis

**DOI:** 10.3389/fphar.2025.1561164

**Published:** 2025-05-16

**Authors:** Pingping Su, Xv Wang, Ruoqian Cai, Chang Yan, Yun Sun

**Affiliations:** Department of Gynaecology, Wenzhou TCM Hospital of Zhejiang Chinese Medical University, Wenzhou, China

**Keywords:** cryptotanshinone, polycystic ovary syndrome, hyperandrogenism, insulin resistance, hyperinsulinemia, metabolic dysfunction

## Abstract

**Background:**

PCOS is a prevalent endocrine disorder characterized by metabolic dysfunctions, including insulin resistance and hyperandrogenism. Cryptotanshinone, a bioactive compound, has shown promise in addressing reproductive abnormalities. However, its therapeutic potential for the management of PCOS has not been sufficiently investigated.

**Purpose:**

The aim of this study is to systematically evaluate the therapeutic efficacy of cryptotanshinone in the treatment of PCOS.

**Methods:**

A comprehensive systematic search was performed across multiple databases, including CNKI, Wanfang Data, VIP, PubMed, and Web of Science, covering studies from their inception through December 2024. The methodological quality of the included studies was assessed using SYRCLE’s Risk of Bias tool. Data synthesis and statistical analysis were conducted using RevMan 5.3 and Stata 17 software.

**Results:**

Seven studies met the inclusion criteria for meta-analysis. Treatment with cryptotanshinone significantly improved several PCOS-related parameters in animal models, as compared to the control group. Notable improvements included reductions in body weight (*P* < 0.00001), ovarian weight (*P* = 0.005), and ovaries quotiety (*P* = 0.0006). Additionally, cryptotanshinone treatment led to significant modulation of serum levels f T (*P* = 0.001), A2 (*P* = 0.02), LH (*P* = 0.0002), LH/FSH ratio (*P* = 0.001), estradiol (*P* = 0.005), and SHBG (*P* = 0.0003). The underlying mechanism may involve the downregulation of CYP17 and AR mRNA and protein expression.

**Conclusion:**

This meta-analysis provides robust evidence supporting the therapeutic efficacy of cryptotanshinone in PCOS. Cryptotanshinone appears to restore both reproductive and metabolic functions, including the regulation of body weight, ovarian morphology, hormone levels, and gene expression. Cryptotanshinone holds significant promise as a potential therapeutic agent for the management of PCOS.

## 1 Introduction

Polycystic ovary syndrome (PCOS) is a prevalent reproductive and endocrine disorder affecting women of reproductive age, with an estimated prevalence ranging from 4% to 21% (Rotterdam ESHRE/ASRM-Sponsored PCOS Consensus Workshop Group, 2004). It is characterized by significant metabolic abnormalities, such as insulin resistance and hyperinsulinemia, as well as reproductive disturbances, including anovulation and hyperandrogenism (Sanchez-Garrido and Tena-Sempere, 2020). These pathophysiological features contribute to a range of clinical manifestations, such as acne, hirsutism, weight instability, and other metabolic abnormalities, and associate with an elevated risk for the development of type 2 diabetes, cardiovascular diseases, and endometrial cancer (Su et al., 2025). Therapeutic interventions aimed at improving insulin sensitivity and reducing androgen levels have shown promise in restoring normal menstrual cycles and promoting ovulation. Consequently, research has increasingly focused on strategies that address both insulin resistance and androgen excess.

**FIGURE 1:**
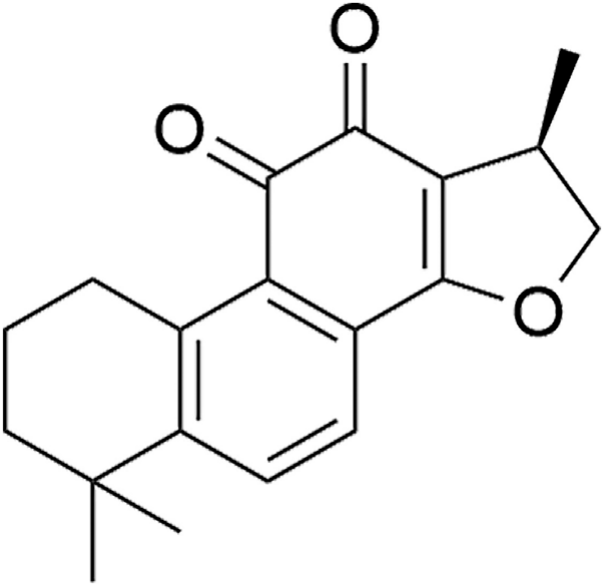
Cryptotanshinone chemical formula.

Cryptotanshinone, a lipophilic diterpenoid compound predominantly derived from species within the Salvia genus, such as Salvia przewalskii Maxim, Salvia tebesana Bunge., and Salvia miltiorrhiza Bunge., has garnered attention due to its potential therapeutic effects (Wu et al., 2020) (Figure 1). Notably, studies have demonstrated that cryptotanshinone possesses both anti-androgenic and insulin-sensitizing properties (Liu et al., 2024; Huang et al., 2014). In addition, it has been shown to exhibit a wide array of pharmacological activities, including anti-tumor (Wang et al., 2024), anti-inflammatory (Ma et al., 2023), cardioprotective (Wang et al., 2021), anti-apoptotic, anti-fibrotic (Wei et al., 2023), and anxiolytic effects (Bian et al., 2023). These findings suggest that cryptotanshinone may play a significant role in enhancing health and preventing disease.

In recent years, substantial research has been conducted to investigate the therapeutic potential of cryptotanshinone in the treatment of PCOS, with particular emphasis on animal models and clinical trials. The compound has demonstrated various beneficial effects in addressing the pathophysiological mechanisms of PCOS, establishing it as a promising candidate for the management of this condition. This review aims to synthesize and critically assess the existing preclinical and clinical data regarding the efficacy of cryptotanshinone in managing PCOS, evaluate its therapeutic mechanisms, and provide insights into its potential clinical application in the treatment of PCOS.

## 2 Materials and methods

### 2.1 Data sources and search strategy

Relevant studies published up to December 2024 were identified through a comprehensive search across multiple databases, without language restrictions. These databases included the Chinese National Knowledge Infrastructure (CNKI), Wanfang Data, Chinese Scientific Journals Database (VIP), PubMed, and Web of Science. The search terms employed were (“Cryptotanshinone”) AND (“Polycystic Ovarian Syndrome” OR “Stein-Leventhal Syndrome” OR “Sclerocystic Ovarian Degeneration” OR “Sclerocystic Ovary Syndrome” OR “PCOS”). A systematic and thorough search strategy was adapted to each database’s specific requirements. The retrieved articles were subsequently imported into NoteExpress software for further management and analysis.

### 2.2 Inclusion criteria

#### 2.2.1 Study design

There were no limitations regarding the language or publication date of the studies included.

#### 2.2.2 Study type

Only randomized controlled animal studies were considered eligible.

#### 2.2.3 Subjects

The studies must involve rats as the experimental subjects.

#### 2.2.4 Experimental model

The studies must utilize a rat model of PCOS.

#### 2.2.5 Intervention

The experimental group received treatment with cryptotanshinone, whereas the control group was not subjected to any intervention. The primary distinction between the experimental and control groups was the administration of cryptotanshinone.

#### 2.2.6 Efficacy evaluation parameters

The efficacy of the intervention was assessed using the following parameters: body weight, ovarian weight, ovaries quotiety, testosterone (T) serum levels, androstenedione (A2) serum levels, luteinizing hormone (LH) serum levels, LH/FSH (follicle stimulating hormone) serum levels, estradiol (E2) serum levels, sex hormone binding globulin (SHBG) serum levels, fasting blood glucose (FBG) serum levels, fasting insulin (FIS) serum levels, Homeostatic Model Assessment of Insulin Resistance (HOMA-IR), protein expression of protein expression of cytochrome P450 17A1 (CYP17), gene expression of CYP17, protein expression of androgen receptor (AR), and gene expression of AR.

### 2.3 Exclusion criteria

#### 2.3.1 Study types

Studies excluded from this review included reviews, conference proceedings, case reports, clinical trials, and *in vitro* studies.

#### 2.3.2 Subjects

Studies involving animal species other than rats were excluded.

#### 2.3.3 Experimental model

Studies that did not employ a PCOS animal model were excluded.

#### 2.3.4 Intervention

Studies that involved the use of drug combinations, rather than cryptotanshinone alone, were excluded.

#### 2.3.5 Outcome measures

Studies were excluded if they did not provide extractable or combinable outcome measures.

#### 2.3.6 Data

Studies with incomplete or missing original data were excluded.

### 2.4 Data extraction

Two independent researchers, both trained uniformly, utilized NoteExpress software to screen the literature. In instances of discrepancies, a third researcher intervened to resolve the conflict. The primary data extracted included the following categories.

#### 2.4.1 General information

This encompassed the title of the study, the name of the first author, year of publication, country, total number of animals, species, age, weight, source of the animals, experimental model, modeling material, the purity and source of cryptotanshinone, and the method of drug administration.

#### 2.4.2 Intervention details

Information gathered in this section included the number of rats, the name of the drug, its concentration, and the duration of exposure for both the experimental and control groups.

#### 2.4.3 Outcomes

In studies where data were solely presented in graphical form, data points were extracted using GetData Graph Digitizer software.

### 2.5 Quality assessment

The SYRCLE Risk of Bias tool (Hooijmans et al., 2014) was employed by two researchers to assess the risk of bias for each study, categorizing the risk as low, high, or unclear according to predefined criteria. Risk of bias plots were generated using RevMan 5.3. Any disagreements between the two researchers were resolved through discussion with a third reviewer.

### 2.6 Statistical analysis

Data analysis was performed using RevMan 5.3 statistical software. For continuous variables, either the mean difference (MD) or the standardized mean difference (SMD) was calculated along with their respective 95% confidence intervals (CIs). In cases where the heterogeneity test yielded *P* > 0.05 and I^2^ < 50%, indicating low or no heterogeneity, a fixed-effects model was applied. Conversely, when *P* < 0.05 and I^2^ > 50% suggested high heterogeneity, a random-effects model was utilized. In instances of substantial heterogeneity, further subgroup analyses were conducted to investigate the sources of variability, based on the specific context of the data.

## 3 Results

### 3.1 Study inclusion

**FIGURE 2:**
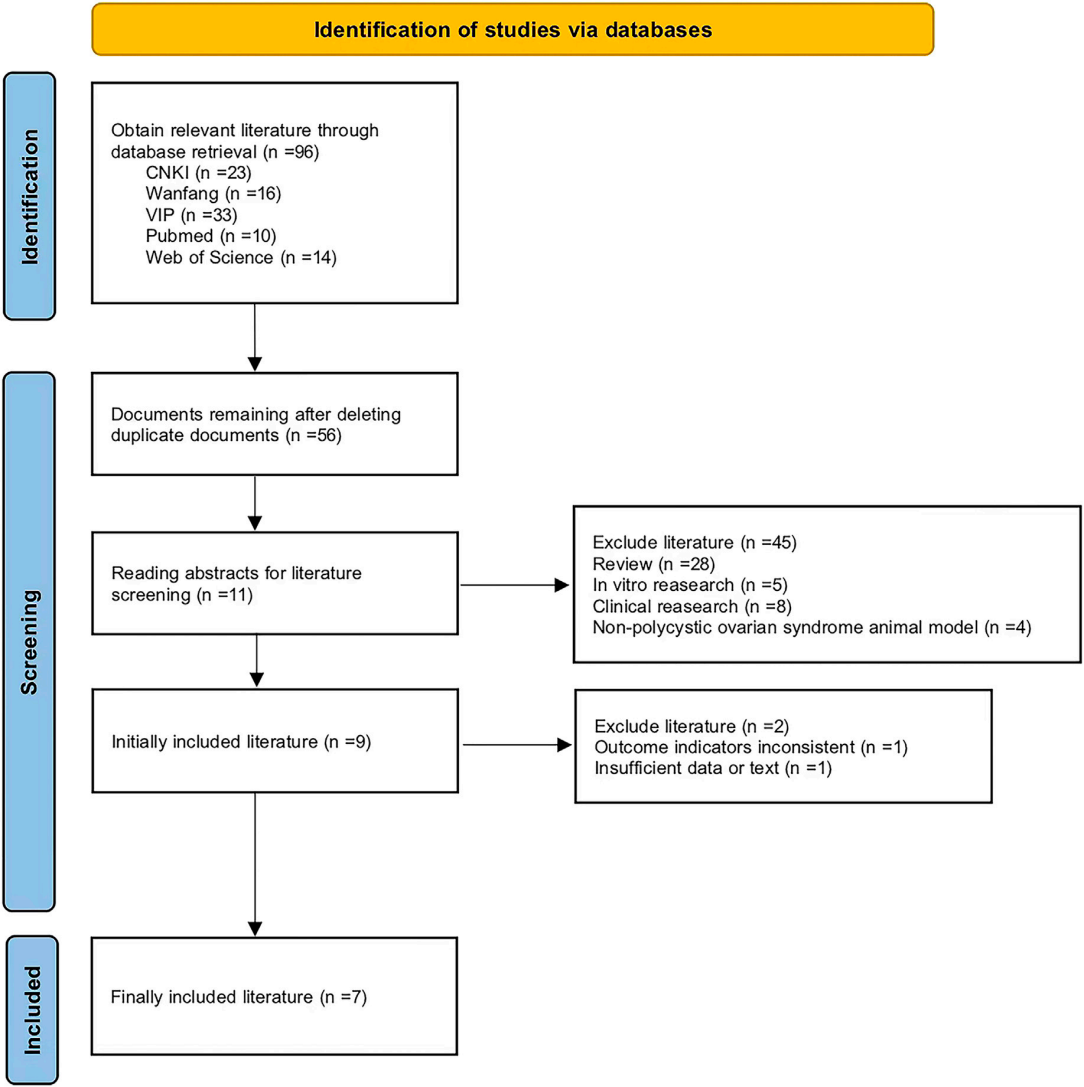
Literature search flow chart.

This investigation was carried out in accordance with the established guidelines set forth by the Preferred Reporting Items for Systematic Reviews and Meta-Analyses (PRISMA) and the Cochrane Collaboration. A total of 96 studies were initially identified. Following the removal of duplicate publications, 56 studies remained. Further exclusions of review articles, clinical and *in vitro* studies, as well as non-PCOS animal model research, resulted in 11 studies. Ultimately, seven studies were deemed eligible for inclusion in the final meta-analysis (Figure 2). The key characteristics of these studies are summarized in Table 1.

**TABLE 1:** Basic characteristics of the included studies.

Study	Country	Animal characteristics	Source of animal	Model	Modeling material	Purity of CRY	Source of CRY	M	E	Administration method	T	Outcome measures
Wang et al. (2015)	China	60 SD rats (50 ± 20 g) 23-day-old	Henan Animal Experiment Center, SCXK (YU) 2010-0002	PCOS	DHEA	98%	Xi’an Haoxuan Biotechnology Co., Ltd.	13 rats, 0.5% CMC	13 rats, 0.5% CMC and 200 mg/kg CRY	Intragastric administration	28d	①④⑥⑦⑩⑪⑫
Yu et al. (2014a)	China	65 Wistar rats (35–40 g) 21-day-old	Shanghai Slack Laboratory Animal Co., Ltd.,SCXK(HU)2007-0003	PCOS	DHEA	99%	Shanghai Ziyi Biological Technology Co., Ltd.	8 rats, 0.01 mL/g normal saline	8 rats, 0.01 mL/g normal saline and 0.027 mg/g/d CRY	Administered orally	28d	①③④⑤⑥⑧⑨⑬⑭⑮⑯
Yang et al. (2020)	China	60 SD rats (170–200 g) 85-day-old	Shanghai Laboratory Animal Center, Co. Ltd.	PCOS	HCG + INS	>98%	Sigma-aldrich	12 rats, saline	12 rats, CRY	Intragastric administration	21d	①②③④⑥
Yu et al. (2014b)	China	50, Wistar rats (35–40 g) 21-day-old	Shanghai Slack Laboratory Animal Co., Ltd.,SCXK(HU)2007-0003	PCOS	DHEA	99%	Shanghai Ziyi Biological Technology Co., Ltd.	12 rats, 0.01 mL/g normal saline	12 rats, 0.01 mL/g normal saline and 0.027 mg/g/d CRY	Administered orally	28d	①③④⑤⑥⑦⑧⑨⑪⑬⑭⑮⑯
Yang et al. (2011)	China	48 Wistar rats 3-month-old	—	PCOS	fetal testosterone treatment	98%	Shanghai First Biochemical Pharmaceutical	12 rats, polysorbate 80 and normal saline	12 rats, polysorbate 80, normal saline, and 0.027 mg/g/d CRY	Administered orally	14d	①②④⑤⑩⑪⑫
Xia et al. (2017)	China	30 SD rats (35–40 g) 21-day-old	Shanghai Laboratory Animal Center of Chinese Academy of Sciences, SCXK-Shanghai 02569	PCOS	DHEA	>98%	Shanghai Yuanye Biological Technology Co., Ltd.	10 rats, normal saline	10 rats, 27 mg/kg CRY	Administered orally	21d	①②③④⑥⑦⑧
Liu et al. (2024)	China	40 SD rats 90-day-old	Laboratory Animal Center of Harbin Medical University, SCXK, 2019001	PCOS	HCG + INS	97%	Shanghai Yuanye Biotechnology Co., Ltd.	12 rats, saline	12 rats, saline and 100 mg/kg CRY	Administered orally	—	①④⑥⑦⑧⑩⑪⑫

Abbreviations: SD, Sprague–Dawley; PCOS, polycystic ovary syndrome; DHEA, dehydroepiandrosterone; HCG, human cho rionic gonadotropin; INS, insulin; CMC, carboxy methylcellulose; CRY, cryptotanshinone; E, experimental group; M, model group; T, time.

Notes: ① body weight; ② ovarian weight; ③ ovaries quotiety; ④ testosterone levels; ⑤ androstenedione levels; ⑥ luteinizing hormone levels; ⑦ luteinizing hormone/follicle stimulating hormone ratio; ⑧ estradiol levels; ⑨ sex hormone binding globulin levels; ⑩ fasting blood glucose levels; ⑪ fasting insulin levels; ⑫ HOMA-IR; ⑬ protein expression of CYP17; ⑭ gene expression of CYP17; ⑮ protein expression of androgen receptor; ⑯ gene expression of androgen receptor.

### 3.2 Risk of bias assessment

**FIGURE 3:**
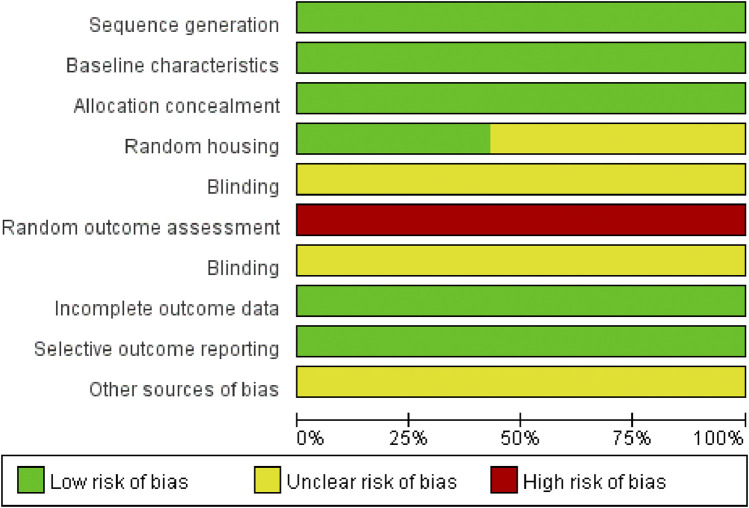
Risk of bias summary: A table summarizing the authors’ judgments regarding each risk of bias item for each individual study.

**FIGURE 4:**
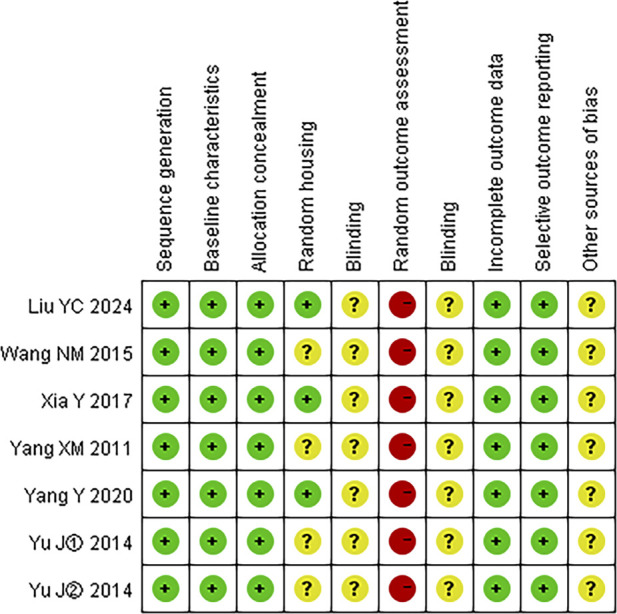
Risk of bias graph: A graphical representation of the distribution of the authors’ judgments across studies for each risk of bias item. Note: “+” represents low risk; “?” represents unclear risk; “−” represents high risk.

The quality of the included studies was evaluated using SYRCLE’s risk of bias tool. Data analysis was performed using RevMan version 5.3 software. The risk of bias summary is presented in Figure 3, while the distribution of risk of bias judgments across the studies is illustrated in Figure 4.

### 3.3 Meta analysis

#### 3.3.1 Body weight

Seven studies (Liu et al., 2024; Wang, 2015; Yu et al., 2014a; Yang et al., 2020; Yu et al., 2014b; Yang et al., 2011; Xia et al., 2017) reported the body weight. They showed that there was heterogeneity (*P* = 0.03, I^2^ = 57%), and the random effect model was adopted. The results showed that cryptotanshinone could effectively reduce body weight (MD = −17.94, 95% CI = [−23.46, −12.42], *P* < 0.00001), and the difference was statistically significant (Table 2).

**TABLE 2:** Meta-analysis results for each outcome indicator.

Outcome indicator	Heterogeneity test results		Effect models	Meta-analysis results		
	I^2^ (%)	*P*		Effect sizes	95% CI	*P*
Body weight	57	0.03	Random	MD = −17.94	[−23.46, −12.42]	<0.00001
Ovarian weight	94	<0.00001	Random	SMD = −5.52	[−9.39, −1.66]	0.005
Ovaries quotiety	77	0.004	Random	SMD = −2.10	[−3.29, −0.90]	0.0006
T levels	88	<0.00001	Random	SMD = −2.08	[−3.34, −0.82]	0.001
A2 levels	94	<0.00001	Random	SMD = −3.98	[−7.29, −0.68]	0.02
LH levels	81	<0.00001	Random	SMD = −1.94	[−2.95, −0.93]	0.0002
LH/FSH	67	0.03	Random	SMD = −1.36	[−2.18, −0.54]	0.001
E2 levels	70	0.02	Random	SMD = −1.29	[−2.20, −0.39]	0.005
SHBG levels	0	1.00	Fixed	MD = 7.12	[3.23, 11.01]	0.0003
FBG levels	95	<0.00001	Random	SMD = −5.91	[−10.58, −1.25]	0.01
FIS levels	91	<0.00001	Random	SMD = −3.77	[−5.96, −1.59]	0.0007
HOMA-IR	100	<0.00001	Random	MD = −4.72	[−8.69, −0.76]	0.02
Protein CYP17	54	0.14	Random	MD = −1.26	[−2.19, −0.32]	0.009
Gene CYP17	0	0.40	Fixed	SMD = −2.24	[−3.27, −1.22]	<0.0001
Protein AR	79	0.03	Random	MD = −2.40	[−4.47, −0.32]	0.02
Gene AR	65	0.09	Random	SMD = −2.85	[−4.86, −0.85]	0.005

#### 3.3.2 Ovarian weight

Three studies (Yang et al., 2020; Yang et al., 2011; Xia et al., 2017) reported the ovarian weight. They showed that there was heterogeneity (*P* < 0.00001, I^2^ = 94%), and the random effect model was adopted. The results showed that cryptotanshinone could effectively reduce ovarian weight (SMD = −5.52, 95% CI = [−9.39, −1.66], *P* = 0.005), and the difference was statistically significant (Table 2).

#### 3.3.3 Ovaries quotiety

Four studies (Yu et al., 2014a; Yang et al., 2020; Yu et al., 2014b; Xia et al., 2017) reported the ovaries quotiety. They showed that there was heterogeneity (*P* = 0.004, I^2^ = 77%), and the random effect model was adopted. The results showed that cryptotanshinone could effectively reduce ovaries quotiety (SMD = −2.10, 95% CI = [−3.29, −0.90], *P* = 0.0006), and the difference was statistically significant (Table 2).

#### 3.3.4 T

Seven studies (Liu et al., 2024; Wang, 2015; Yu et al., 2014a; Yang et al., 2020; Yu et al., 2014b; Yang et al., 2011; Xia et al., 2017) reported the T serum levels. They showed that there was heterogeneity (*P* < 0.00001, I^2^ = 88%), and the random effect model was adopted. The results showed that cryptotanshinone could effectively reduce the T serum levels (SMD = −2.08, 95% CI = [−3.34, −0.82], *P* = 0.001), and the difference was statistically significant (Table 2).

#### 3.3.5 A2

Three studies (Yu et al., 2014a; Yu et al., 2014b; Yang et al., 2011) reported the A2 serum levels. They showed that there was heterogeneity (*P* < 0.00001, I^2^ = 94%), and the random effect model was adopted. The results showed that cryptotanshinone could effectively reduce the A2 serum levels (SMD = −3.98, 95% CI = [−7.29, −0.68], *P* = 0.02), and the difference was statistically significant (Table 2).

#### 3.3.6 LH

Six studies (Liu et al., 2024; Wang, 2015; Yu et al., 2014a; Yang et al., 2020; Yu et al., 2014b; Xia et al., 2017) reported the LH serum levels. They showed that there was heterogeneity (*P* < 0.00001, I^2^ = 81%), and the random effect model was adopted. The results showed that cryptotanshinone could effectively reduce the LH serum levels (SMD = −1.94, 95% CI = [−2.95, −0.93], *P* = 0.0002), and the difference was statistically significant (Table 2).

#### 3.3.7 LH/FSH

Four studies (Liu et al., 2024; Wang, 2015; Yu et al., 2014b; Xia et al., 2017) reported the LH/FSH serum levels. They showed that there was heterogeneity (*P* = 0.03, I^2^ = 67%), and the random effect model was adopted. The results showed that cryptotanshinone could effectively reduce the LH/FSH serum levels (SMD = −1.36, 95% CI = [−2.18, −0.54], *P* = 0.001), and the difference was statistically significant (Table 2).

#### 3.3.8 E2

Four studies (Liu et al., 2024; Yu et al., 2014a; Yu et al., 2014b; Xia et al., 2017) reported the E2 serum levels. They showed that there was heterogeneity (*P* = 0.02, I^2^ = 70%), and the random effect model was adopted. The results showed that cryptotanshinone could effectively reduce the E2 serum levels (SMD = −1.29, 95% CI = [−2.20, −0.39], *P* = 0.005), and the difference was statistically significant (Table 2).

#### 3.3.9 SHBG

Two studies (Yu et al., 2014a; Yu et al., 2014b) reported the SHBG serum levels. They showed that there was heterogeneity (*P* = 1.00, I^2^ = 0%), and the random effect model was adopted. The results showed that cryptotanshinone could effectively reduce the SHBG serum levels (MD = 7.12, 95% CI = [3.23, 11.01], *P* = 0.0003), and the difference was statistically significant (Table 2).

#### 3.3.10 FBG

Three studies (Liu et al., 2024; Wang, 2015; Yang et al., 2011) reported the serum levels of FBG. They showed that there was heterogeneity (*P* < 0.00001, I^2^ = 95%), and the random effect model was adopted. The results showed that cryptotanshinone could effectively reduce the serum levels of FBG (SMD = −5.91, 95% CI = [−10.58, −1.25], *P* = 0.01), and the difference was statistically significant (Table 2).

#### 3.3.11 FIS

Four studies (Liu et al., 2024; Wang, 2015; Yu et al., 2014b; Yang et al., 2011) reported the serum levels of FIS. They showed that there was heterogeneity (*P* < 0.00001, I^2^ = 91%), and the random effect model was adopted. The results showed that cryptotanshinone could effectively reduce the serum levels of FIS (SMD = −3.77, 95% CI = [−5.96, −1.59], *P* = 0.0007), and the difference was statistically significant (Table 2).

#### 3.3.12 HOMA-IR

Three studies (Liu et al., 2024; Wang, 2015; Yang et al., 2011) reported the serum levels of HOMA-IR. They showed that there was heterogeneity (*P* < 0.00001, I^2^ = 100%), and the random effect model was adopted. The results showed that cryptotanshinone could effectively reduce the serum levels of HOMA-IR (MD = −4.72, 95% CI = [−8.69, −0.76], *P* = 0.02), and the difference was statistically significant (Table 2).

#### 3.3.13 Protein CYP17

Two studies (Yu et al., 2014a; Yu et al., 2014b) reported the expression of protein CYP17. They showed that there was heterogeneity (*P* = 0.14, I^2^ = 54%), and the random effect model was adopted. The results showed that cryptotanshinone could effectively reduce the expression of protein CYP17 (MD = −1.26, 95% CI = [−2.19, −0.32], *P* = 0.009), and the difference was statistically significant (Table 2).

#### 3.3.14 Gene CYP17

Two studies (Yu et al., 2014a; Yu et al., 2014b) reported the expression of gene CYP17. They showed that there was heterogeneity (*P* = 0.40, I^2^ = 0%), and the random effect model was adopted. The results showed that cryptotanshinone could effectively reduce the expression of gene CYP17 (SMD = −2.24, 95% CI = [−3.27, −1.22], *P* < 0.0001), and the difference was statistically significant (Table 2).

#### 3.3.15 Protein AR

Two studies (Yu et al., 2014a; Yu et al., 2014b) reported the expression of protein AR. They showed that there was heterogeneity (*P* = 0.03, I^2^ = 79%), and the random effect model was adopted. The results showed that cryptotanshinone could effectively reduce the expression of protein AR (MD = −2.40, 95% CI = [−4.47, −0.32], *P* = 0.02), and the difference was statistically significant (Table 2).

#### 3.3.16 Gene AR

Two studies (Yu et al., 2014a; Yu et al., 2014b) reported the expression of gene AR. They showed that there was heterogeneity (*P* = 0.09, I^2^ = 65%), and the random effect model was adopted. The results showed that cryptotanshinone could effectively reduce the expression of gene AR (SMD = −2.85, 95% CI = [−4.86, −0.85], *P* = 0.005), and the difference was statistically significant (Table 2).

### 3.4 Sensitivity analysis

#### 3.4.1 Ovarian weight

**FIGURE 5:**
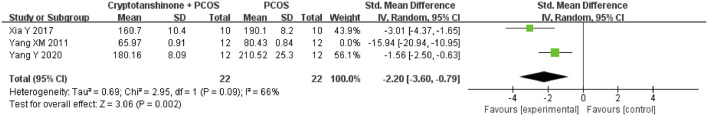
Forest plot of ovarian weight by leave-one out analysis.

A sensitivity analysis of ovarian weight was performed using a leave-one-out analysis. The study by Yang et al. (2011) emerged as the primary source of heterogeneity, with the I^2^ statistic reducing from 94% to 66% upon its exclusion. Based on a review of the original studies, we hypothesize that variations in the animal species used contributed to this heterogeneity. Specifically, Yang et al. (2011) employed Wistar rats, while Xia et al. (2017) and Yang et al. (2020) used SD rats in their respective studies. Despite these differences, the results consistently support the conclusion that cryptotanshinone treatment reduces ovarian weight in PCOS rats (Figure 5).

#### 3.4.2 A2

**FIGURE 6:**
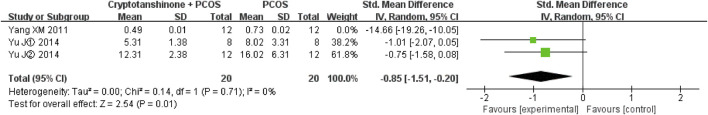
Forest plot of A2 by leave-one out analysis.

A sensitivity analysis was conducted on A2 serum levels using the leave-one-out analysis. The study by Yang et al. (2011) was identified as the principal contributor to heterogeneity, with the I^2^ statistic decreasing from 94% to 0% upon its removal. Upon further examination of the original texts, we speculate that the age differences of the rats in the studies may account for the observed heterogeneity. Specifically, Yang et al. (2011) study utilized 3-month-old rats, whereas Yu et al. (2014a); Yu et al. (2014b) study involved rats aged 21 days. Nevertheless, the findings remain consistent, suggesting that cryptotanshinone administration effectively lowers A2 serum levels in PCOS rats (Figure 6).

#### 3.4.3 FBG

**FIGURE 7:**
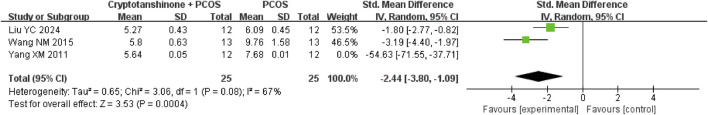
Forest plot of FBG by leave-one out analysis.

We performed a sensitivity analysis on FBG levels through leave-one-out analysis. The study by Yang et al. (2011) was the major source of heterogeneity, with the I^2^ statistic decreasing from 95% to 67% after its exclusion. Differences in the modeling periods across studies were identified as a potential cause of heterogeneity. In Yang et al. (2011) study, the PCOS model was induced during the fetal period, while Liu et al. (2024) and Wang (2015) studies employed a postnatal modeling approach. Despite these methodological differences, the overall results provide strong evidence that cryptotanshinone treatment reduces FBG levels in PCOS rats (Figure 7).

#### 3.4.4 Ovaries quotiety

**FIGURE 8:**
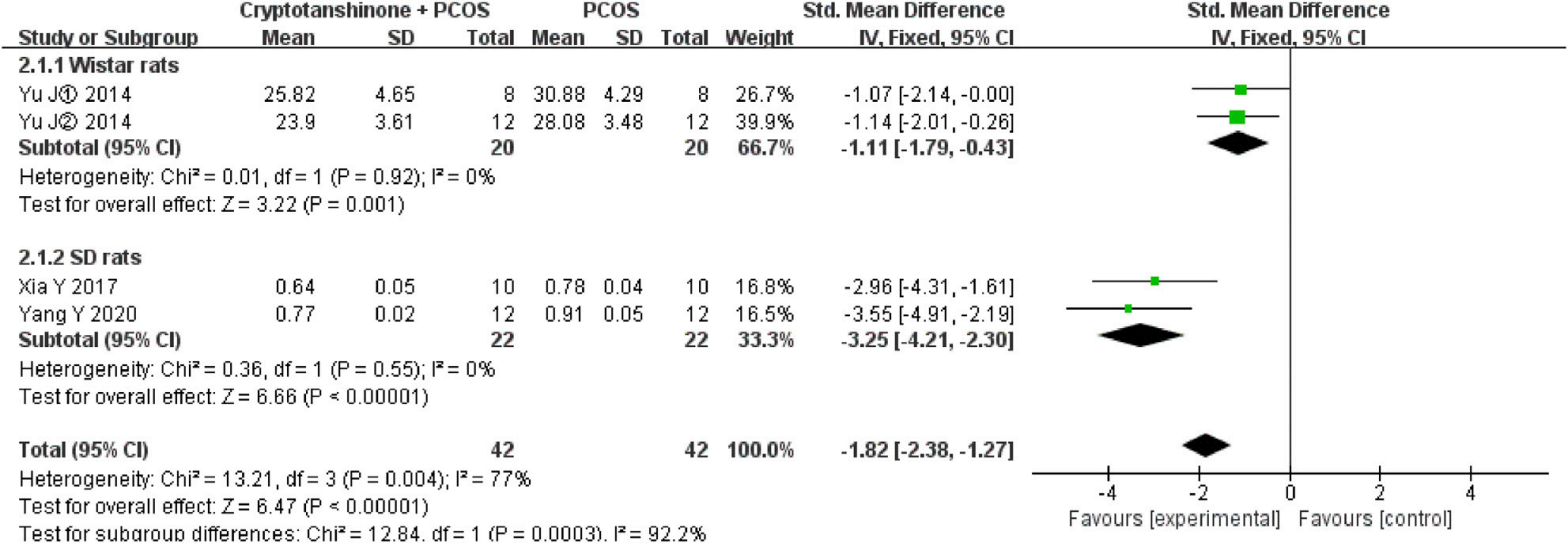
Forest plot of ovaries quotiety divided into subgroups based on animal breed.

Subgroup analysis based on animal breed revealed that heterogeneity was significantly reduced when the analysis was restricted to SD rats or Wistar rats, with I^2^ decreasing to 0%. Additionally, the studies conducted by Yu et al. (2014a); Yu et al. (2014b) were reported by the same research team. They were published in 2014, utilizing cryptotanshinone with a purity of 99%. The rats were supplied by Shanghai Slack Laboratory Animal Co., Ltd. In contrast, the studies reported by Xia et al. (2017) and Yang et al. (2020) were conducted by separate research teams. Xia et al. (2017) study was published in 2017, with rats supplied by Shanghai Laboratory Animal Center of the Chinese Academy of Sciences, while Yang et al. (2020) study was published in 2020, with rats supplied by Shanghai Laboratory Animal Center Co., Ltd. The cryptotanshinone used in both Xia Y and Yang Y’s studies had a purity greater than 98%. However, the results robustly support the conclusion that cryptotanshinone treatment effectively reduces ovaries quotiety in PCOS rats (Figure 8).

#### 3.4.5 T

**FIGURE 9:**
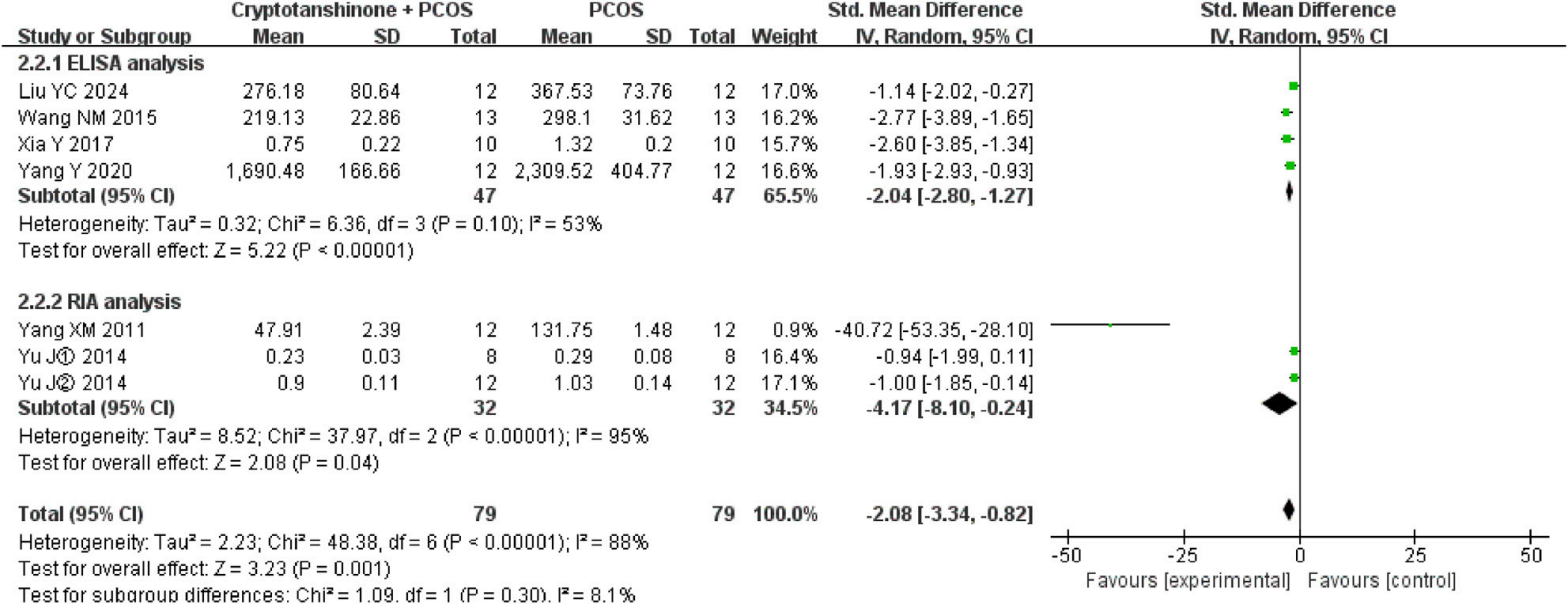
Forest plot of T levels divided into subgroups based on the method of serum T assessment.

Subgroup analysis was performed based on the method of serum T assessment. The heterogeneity test showed a reduction in I^2^ to 53% when enzyme-linked immunosorbent assay (ELISA) was used, whereas the I^2^ statistic increased to 95% when radioimmunoassay (RIA) was employed. Regardless of the analytical method, the findings consistently indicate that cryptotanshinone effectively reduces serum T levels in PCOS rats (Figure 9).

#### 3.4.6 LH

**FIGURE 10:**
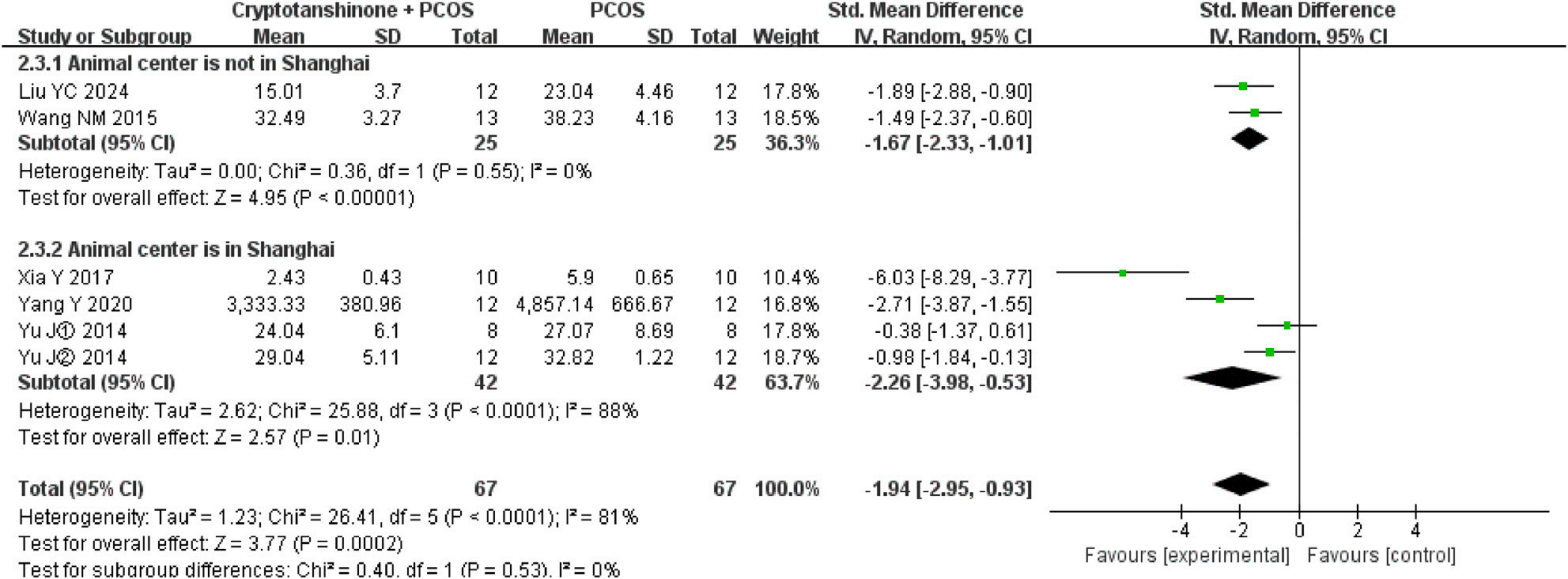
Forest plot of LH levels divided into subgroups based on the geographical location of the animal procurement center.

A subgroup analysis based on the geographical location of the animal procurement center revealed significant differences in heterogeneity. When rats were sourced from Shanghai, which is geographically proximal, I^2^ decreased to 0%, whereas procurement from centers in Henan or Harbin, which are more distant, resulted in an I^2^ value of 88%. Despite these regional differences, cryptotanshinone treatment was consistently shown to effectively reduce LH serum levels in PCOS rats (Figure 10).

#### 3.4.7 E2

**FIGURE 11:**
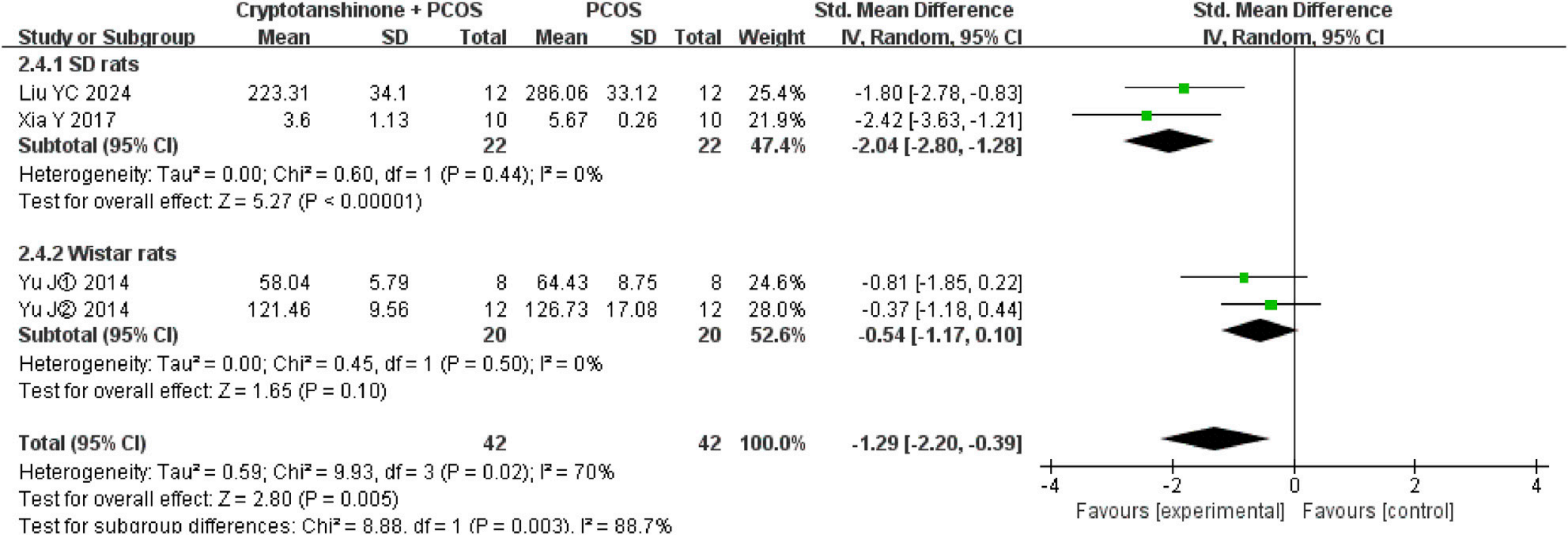
Forest plot of E2 levels divided into subgroups based on animal breed.

A subgroup analysis based on animal breed demonstrated a significant reduction in heterogeneity, with I^2^ decreasing to 0% for both SD and Wistar rats. These results further support the conclusion that cryptotanshinone administration is effective in reducing E2 serum levels in PCOS rats (Figure 11).

#### 3.4.8 FIS

**FIGURE 12:**
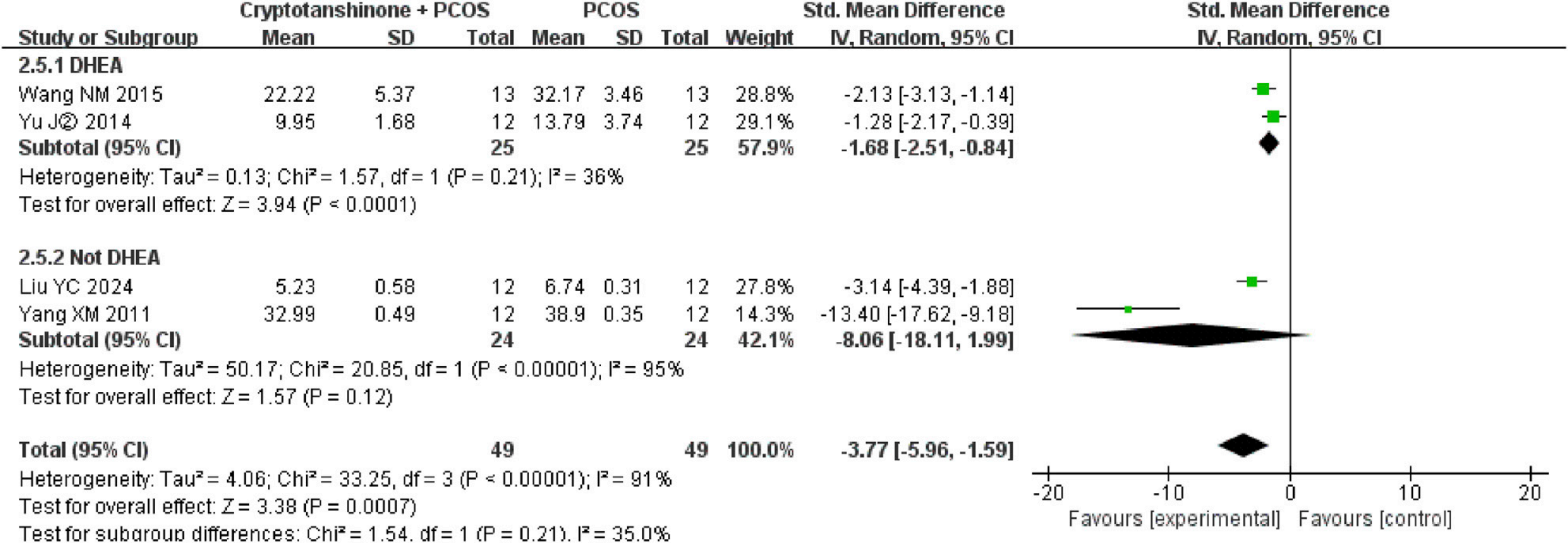
Forest plot of FIS levels divided into subgroups based on the modeling agent.

A subgroup analysis based on the modeling agent used indicated considerable heterogeneity. The I^2^ statistic decreased to 36% when DHEA was used as the modeling agent, whereas it increased to 95% when testosterone, HCG, and INS were used. Nonetheless, the results consistently demonstrate that cryptotanshinone treatment effectively reduces FIS serum levels in PCOS rats (Figure 12).

### 3.5 Publication bias

**FIGURE 13:**
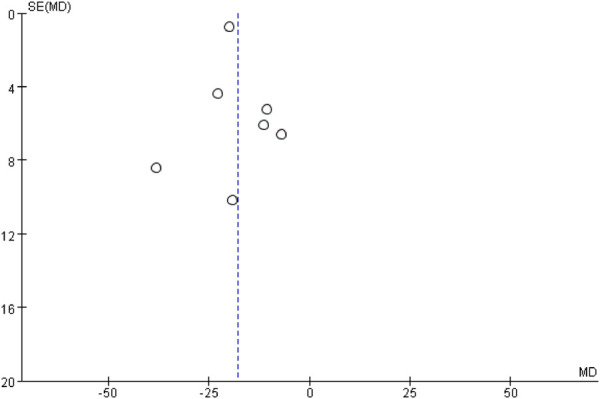
Funnel chart of body weight.

The publication bias of body weight was assessed by the funnel plot. The analysis revealed a generally symmetrical distribution, although some degree of publication bias was evident. However, the Egger’s test results showed no significant bias (*P* = 0.593) (Figure 13).

## 4 Discussion

PCOS is a multifactorial disorder characterized by reproductive dysfunction and metabolic abnormalities, which significantly impact women’s reproductive health and quality of life. The reproductive disturbances in PCOS are primarily manifested by ovarian dysfunction, including anovulation and excessive androgen production, while the metabolic disturbances are marked by insulin resistance and hyperinsulinemia.

Our meta-analyses have highlighted the potential therapeutic benefits of cryptotanshinone in the management of PCOS, which include the following key effects: (1) Weight regulation: Cryptotanshinone has been shown to effectively reduce body weight in PCOS animal models, possibly through the enhancement of insulin sensitivity and the reduction of hyperglycemia, contributing to overall metabolic health. (2) Ovarian environment optimization: Treatment with cryptotanshinone significantly reduces both ovarian weight and the ovaries quotiety, suggesting its potential to improve the pathological changes in the ovaries and restore normal ovulatory function. (3) Hormonal modulation: Cryptotanshinone effectively lowers serum levels of T, A2, LH, and the LH/FSH ratio, which may aid in the restoration of normal hypothalamic-pituitary-ovarian (H-P-O) axis function and mitigate hyperandrogenism. Furthermore, the compound reduces E2 levels, which is important for balancing estrogen and androgen levels. Notably, cryptotanshinone increases the levels of SHBG, which not only modulates the bioavailability of androgens but also mitigates the adverse effects of free androgens, alleviating hyperandrogenic symptoms. (4) Metabolic improvement: Cryptotanshinone has been shown to significantly improve FBG, FIS levels, and insulin resistance, as indicated by the HOMA-IR index, suggesting its potential in improving insulin sensitivity and glycemic control—crucial for addressing metabolic complications in PCOS. (5) Gene and protein regulation: Cryptotanshinone downregulates the expression of CYP17 and AR proteins, along with their respective genes, thereby suggesting its therapeutic effects through inhibition of the androgen synthesis pathway and suppression of androgen signaling.

The neuroendocrine disruptions in PCOS include an increased frequency of gonadotropin-releasing hormone (GnRH) pulses, which in turn elevates LH secretion (Silva and Campbell, 2022). Elevated LH levels act on the thecal cells of the ovaries, promoting androgen synthesis (Rosenfield and Ehrmann, 2016). In the context of persistent high-frequency LH pulses, there is an upregulation of CYP17 mRNA expression in ovarian follicular cells, leading to increased activity of key enzymes in the androgen biosynthesis pathway—17-α-hydroxylase and 17,20-lyase (Jahromi et al., 2016; Diamanti-Kandarakis et al., 2008). These enzymes facilitate the conversion of pregnenolone to 17-hydroxypregnenolone and further to A2, respectively (Yu et al., 2014b). Concurrently, small follicles in the ovaries produce estrone (E1) and E2, which, through aromatase activity, act on the hypothalamus and pituitary gland, establishing a positive feedback loop that exacerbates LH secretion. This imbalance in the LH/FSH ratio disrupts normal ovarian function and contributes to the hyperandrogenism characteristic of PCOS. Ultimately, this feedback loop perpetuates the pathophysiology of PCOS, exacerbating both reproductive and metabolic disturbances.

Hyperinsulinemia exacerbates PCOS by increasing the sensitivity of ovarian thecal cells to LH through upregulation of LH receptors. This facilitates the overproduction of androgens in the ovaries (Cara and Rosenfield, 1988; Zhang et al., 2000). The synergistic interaction between LH and insulin further amplifies the expression of CYP17, thereby increasing the activity of the cytochrome P450 enzyme system, particularly the 17-α-hydroxylase and 17,20-lyase activities (Rosenfield and Ehrmann, 2016; Cara and Rosenfield, 1988; Toprak et al., 2001). These enzymes are pivotal in the androgen biosynthesis pathway, catalyzing the conversion of steroid precursors to androgens. Hence, insulin contributes to androgen overproduction not only by enhancing LH-induced stimulation but also by directly increasing the enzymatic activity in the steroidogenic pathway.

SHBG plays a critical role in modulating the bioavailability of sex hormones by binding tightly to circulating androgens and estrogens (Hammond et al., 2012). In PCOS, elevated androgen levels are often associated with decreased SHBG concentrations, a phenomenon exacerbated by hyperinsulinemia. Insulin resistance and hyperinsulinemia reduce SHBG synthesis in the liver, further promoting hyperandrogenism and creating a vicious cycle (Bourebaba et al., 2022; Wang et al., 2023). Elevated LH levels may also directly suppress SHBG production, thereby diminishing its ability to bind free testosterone and further exacerbating androgen excess. The reduction in SHBG levels thus contributes to the pathophysiology of PCOS by amplifying the effects of circulating free androgens.

Androgenic actions are primarily mediated through the nuclear AR, which is essential for normal follicular development and ovulation (Cheng et al., 2013). Dysregulation of AR activity, particularly through upregulation, has been implicated in the pathogenesis of PCOS, where excessive androgen levels lead to increased follicular arrest and reduced mature follicle formation (Walters et al., 2019). Although androgens are essential for early follicular development, their excess can inhibit the expression of genes critical for cumulus expansion and oocyte maturation, thereby preventing the transition from pre-antral to antral follicle stages. This results in the accumulation of immature follicles and impaired ovulation (Liao et al., 2022). Therefore, maintaining a balance in androgen levels and AR activity is vital for ensuring normal ovarian function. Targeting the AR signaling pathway may represent a potential therapeutic approach for restoring follicular development and improving reproductive outcomes in women with PCOS.

PCOS is characterized by profound endocrine and metabolic disturbances, including hyperandrogenism, insulin resistance, and hyperinsulinemia (Su et al., 2025). This meta-analysis systematically evaluates the therapeutic effects of cryptotanshinone, demonstrating its multifaceted therapeutic potential via modulation of the “androgen-insulin” interaction network. The key findings include: (1) Bidirectional regulation of hyperandrogenism: Cryptotanshinone significantly decreases serum T levels, with its mechanism likely involving the inhibition of CYP17, a critical enzyme in ovarian steroidogenesis. Overexpression of CYP17 in the follicular membrane cells of PCOS rats limits androgen production, and cryptotanshinone’s modulation of the LH/FSH ratio suggests its involvement in the regulation of the H-P-O axis. By restoring the physiological pulsatile secretion of gonadotropins, cryptotanshinone may compensate for the inadequate regulation of the H-P-O axis by conventional ovulation-inducing agents like clomiphene. (2) Precise regulation of sex hormone balance: The reduction in E2 levels reflects cryptotanshinone’s impact on aromatase activity. Elevated aromatase activity in the ovarian interstitium of PCOS rats disrupts local estrogen production, exacerbating LH secretion imbalance through a positive feedback mechanism. Moreover, cryptotanshinone promotes the synthesis of SHBG, which not only mitigates free testosterone activity but may also improve liver insulin sensitivity—considering that hyperinsulinemia inhibits SHBG synthesis. (3) Multisystem metabolic regulation: Cryptotanshinone improves FBG and HOMA-IR, suggesting its potential as an insulin sensitizer. High androgen levels exacerbate insulin resistance through AR signaling, while hyperinsulinemia promotes androgen synthesis. Cryptotanshinone exhibits potential to break this pathological cycle. (4) Translational medicine innovation: The triple regulatory effects of cryptotanshinone on CYP17 (androgen synthesis), AR (androgen signaling), and HOMA-IR (insulin resistance) offer a promising therapeutic strategy targeting the core pathogenic network of PCOS.

In conclusion, cryptotanshinone modulates the endocrine and metabolic profiles of PCOS rats through multiple mechanisms, including the bidirectional regulation of hyperandrogenism, the precise modulation of sex hormone equilibrium, and the coordinated metabolic regulation across multiple organs. These effects underscore its distinct therapeutic advantages and potential. The findings presented herein provide a robust foundation for further investigation into the therapeutic application of cryptotanshinone in the management of PCOS.

### 4.1 Shortcomings and prospects

The existing literature on cryptotanshinone for PCOS has notable limitations that warrant attention in future research. First, there is a significant risk of detection bias due to the absence of random outcome assessments in current studies, which diminishes the reliability and reproducibility of findings. To mitigate potential biases and enhance the robustness of results, future investigations should incorporate more rigorous randomization and blinding methodologies. Second, the relatively small sample sizes in current studies hinder the broader applicability of the conclusions, highlighting the need for larger, more diverse participant cohorts to strengthen the generalizability of the findings. Third, while prior research suggests that cryptotanshinone may offer therapeutic benefits for PCOS, the precise mechanisms underlying its action remain insufficiently understood. Comprehensive mechanistic studies are essential to elucidate the pathways through which cryptotanshinone exerts its effects. Finally, optimizing the dosing regimen of cryptotanshinone is crucial for maximizing its therapeutic potential. Further exploration into the optimal timing, dosage, and duration of treatment is necessary, and systematic experimentation will be essential in establishing evidence-based protocols that can guide clinical application.

## 5 Conclusion

This study demonstrates that cryptotanshinone exerts significant therapeutic effects on the pathophysiological processes of PCOS through multi-target interventions. In the metabolic regulation, cryptotanshinone reduces body weight in PCOS rats, enhances insulin sensitivity, and improves FBG levels, thereby alleviating insulin resistance. In the ovarian function restoration, cryptotanshinone decreases ovarian weight and ovaries quotiety, promotes follicular development, and facilitates the recovery of the ovulation cycle. In the hormonal balance modulation, cryptotanshinone lowers serum levels of T, A2, and LH, adjusts the LH/FSH ratio, restores the function of the H-P-O axis, and regulates E2 levels, thereby maintaining overall sex hormone balance. And in the androgen signaling inhibition, cryptotanshinone upregulates SHBG levels, thereby reducing the bioavailability of androgens, and directly inhibits CYP17 and AR expression, thereby blocking androgen synthesis and associated signaling pathways. Additionally, the beneficial effects of cryptotanshinone on FBG, FIS levels and the HOMA-IR index further support its potential role in preventing the metabolic complications commonly associated with PCOS. This study provides robust experimental evidence for the mechanisms underlying the use of cryptotanshinone as a novel therapeutic strategy for treating PCOS, with its multi-target mode of action offering promising new avenues for clinical intervention in this disorder.

## Data Availability

The original contributions presented in the study are included in the article/Supplementary Material, further inquiries can be directed to the corresponding author.
